# Role of the CHA_2_DS_2_-VASc score in predicting hospital stay and 90-day readmission among patients with atrial fibrillation in Syria

**DOI:** 10.1177/03000605251314807

**Published:** 2025-02-08

**Authors:** Ibrahim Antoun, Alamer Alkhayer, Alkassem Alkhayer, Yaman Mahfoud, Ahmed Kotb, Riyaz Somani, G André Ng, Mustafa Zakkar

**Affiliations:** 1 Department of Cardiovascular Sciences, University of Leicester, Leicester, UK; 2 Faculty of Medicine, University of Aleppo, Aleppo, Syria; 3 University of Tishreen Hospital, Latakia, Syria; 4 NIHR Leicester Biomedical Research Centre, Leicester, UK; 5 Department of Cardiology, University Hospitals of Leicester NHS Trust, Glenfield Hospital, Leicester, UK; 6 Department of Cardiac Surgery, University Hospitals of Leicester NHS Trust, Glenfield Hospital, Leicester, UK; 7 Faculty of Medicine, University of Damascus, Damascus, Syria

**Keywords:** Atrial fibrillation, mortality, readmission, CHA_2_DS_2_-VASc, Syria, conflict

## Abstract

**Objectives:**

We assessed the CHA_2_DS_2_-VASc score for predicting hospital readmission risk and length of stay (LOS) in patients admitted with primary atrial fibrillation (AF).

**Methods:**

This retrospective cohort study included patients with index admission for AF to Latakia’s tertiary center (May 2021–November 2023). Patients were followed 90 days to assess readmission. CHA_2_DS_2_-VASc was correlated with 90-day readmission, inpatient all-cause mortality, and LOS during index admission.

**Results:**

In total, 717 patients were included; 320 (45%) were readmitted to the hospital within 90 days (58% men, 65% aged <65 years). Inpatient mortality was 4%; the median LOS was 2 days. There was an increase in the incident rate ratio (IRR) of LOS starting from a CHA_2_DS_2_-VASc of 2 (IRR: 2, 95% confidence interval [CI]: 1.7–2.2) to a score of >6 (IRR: 5, 95% CI: 1.8–10.7), compared with a score of 0. There was an incremental increase in the hazard ratio (HR) of readmission from a score of 1 (HR: 2.3, 95% CI: 1.3–4.1) to a score of >6 (HR: 41, 95% CI: 31–72) compared with a CHA_2_DS_2_-VASc of 0.

**Conclusion:**

CHA_2_DS_2_-VASc could predict 90-day hospital readmission and LOS during the index admission in patients admitted with primary AF.

## Introduction

Atrial fibrillation (AF) is the most common type of arrhythmia worldwide, and its prevalence in low-to-middle-income countries is underestimated.^1^ AF in the developed world is well studied. However, there are limited data on AF management and demographics in the Middle East, with only four data registries.^2^ AF-related research in Arab countries contributes only 0.7% to AF research worldwide.^3^

Syria has been experiencing conflict since 2011. The country has been deprived of health care funding and resources, particularly exacerbated during the cholera and COVID-19 outbreaks.^4,5^ Therefore, fewer than 50% of Syria’s hospitals operate at usual performance levels, with more than half of its health care workforce forced to leave the country owing to conflict.^6^ AF management in hospitals during the current conditions of economic and political turmoil is unclear, with a lack of published inpatient figures and outcomes originating from Syrian health care facilities. In the context of these resource limitations, a real-world description of current AF care in Syria can help with managing and allocating resources by recognizing remediable deficiencies and, more importantly, practical and reasonable solutions that can be enforced.^7^ Although late advances in AF management have enhanced the AF burden and symptom control, hospital readmission rates continue to increase and have been a primary source of AF‐related financial constraints on health care economies around the world. Particularly for Syria, following up on patients after initial admissions related to AF is highly challenging owing to limited resources and damaged infrastructure.^6^ The CHA_2_DS_2_-VASc (congestive heart failure [CHF]; hypertension; age ≥75 years [doubled]; type-2 diabetes; previous stroke or transient ischemic attack [doubled]; vascular disease; sex category; and age 65–75 years) score has been recommended for thromboembolic risk assessment in patients with AF.^8^ The score represents the clustering of risk factors associated with higher cardiovascular risk.

The CHA_2_DS_2_-VASc score is integral to clinical practice guidelines worldwide, serving as a cornerstone for decisions regarding anticoagulation therapy to prevent stroke in patients with AF.^9^ The score’s simplicity, incorporating readily available clinical parameters, makes it a practical tool for routine use in diverse health care settings. Beyond its traditional role in thromboembolic risk assessment, emerging research has highlighted the score’s utility in predicting broader cardiovascular outcomes, including CHF,^10,11^ myocardial infarction,^12^ and even mortality, in both AF and non-AF populations.^13^ This wider application underscores its potential as a versatile risk stratification tool.

Given the limited health care resources and infrastructure challenges in conflict-affected regions such as Syria, the CHA_2_DS_2_-VASc score offers a valuable and pragmatic approach to evaluating cardiovascular risk and predicting clinical outcomes. The 90-day readmission rate and predictors of hospital readmission and length of stay (LOS) have been studied in the United States (US) but not in a developing country undergoing conflict.^14^ In this study, we aimed to build on this growing evidence by assessing the CHA_2_DS_2_-VASc score’s predictive value for inpatient outcomes and 90-day readmissions in a Syrian cohort with AF. Understanding its utility in such a setting could inform practical interventions to improve patient care and resource allocation in similar resource-constrained environments.

## Methods

This single-center retrospective observational cohort study was conducted at Tishreen University Hospital in Latakia, Syria. Inclusion criteria were patients over 18 years old presenting to the emergency department between 1 May 2021 and 1 November 2023 and treated with AF as the primary diagnosis during the initial admission. Exclusion criteria were patients younger than 18 years with incomplete demographic or clinical data or an AF diagnosis secondary to reversible causes such as hyperthyroidism or alcohol intoxication. To assess hospital readmission, patients were followed for 90 days following discharge from their index admission.

Data were collected retrospectively using the hospital’s paper records. Patient information was extracted, including demographics, medical history, CHA_2_DS_2_-VASc score components, and admission details. Two independent researchers cross-verified the data extraction process to ensure data completeness and accuracy. Any discrepancies were resolved by consensus with the supervising researcher. Additionally, index admission diagnoses were confirmed using a standardized protocol involving discussions with the on-call cardiology consultant to ensure diagnostic consistency.

Follow-up data for 90-day readmissions were obtained from the hospital paper charts, which were cross-checked with the admission database to document readmissions. Owing to resource limitations in the health care system, no further data were available for patients who did not return to the hospital within the follow-up period. However, the completeness of follow-up data was estimated to be high because Tishreen University Hospital is one of the main referral centers in the region.

The study’s primary outcomes included inpatient all-cause mortality, LOS during the index admission, and the 90-day readmission rate. In secondary analysis, we explored the correlation between the CHA_2_DS_2_-VASc score and 90-day readmission trends. Patient details have been de-identified. The research reported in this article adhered to the Declaration of Helsinki. The institutional ethics committee of Tishreen University Hospital reviewed and approved this study protocol (reference 282/A; date of approval 02/04/2021). The project was conducted as part of an audit approved by the hospital board and involved prospective analysis of retrospectively collected anonymized data. Therefore, the hospital board waived the need for informed consent. The reporting of this study conforms to the Strengthening the Reporting of Observational Studies in Epidemiology (STROBE) guidelines.^15^

## Statistical analysis

The Kruskal–Wallis test or Student *t*-test was used to compare continuous variables depending on the normality of the distribution, and the Pearson’s χ^2^ test or Fisher’s exact test was used for categorical data. Continuous variables are expressed as median and interquartile range (IQR); categorical variables are presented as count and percentage. Incidence rate ratios (IRRs) were calculated using negative binomial regression to assess the relationship between the CHA_2_DS_2_-VASc score and (LOS) during index admissions. Hazard ratios (HRs) for 90-day readmissions were estimated using Cox proportional hazards regression models, with time-to-event data censored at 90 days post-discharge. Kaplan–Meier curves were constructed to assess the cumulative incidence of 90-day readmission. Logistic regression was used to assess the odds ratio (OR) associated with incremental increases in the CHA_2_DS_2_-VASc score to evaluate inpatient mortality. A two-sided P-value <0.05 was considered statistically significant throughout the analysis. For multiple comparisons, adjustments were made to control for type I errors, where applicable. All analyses were performed using GraphPad Prism version 10.0 for Mac (GraphPad Software Inc., San Diego, CA, USA).

## Results

**Figure 1.**
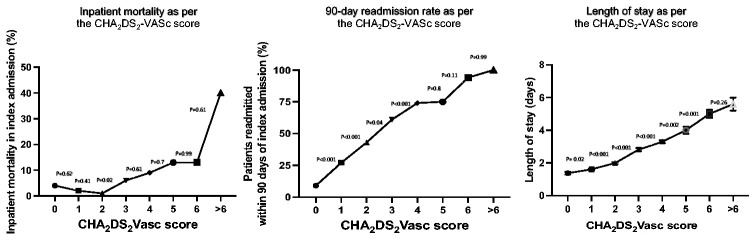
Role of the CHA_2_DS_2_-VASc score in predicting inpatient mortality, hospital length of stay, and 90-day readmission.

**Figure 2.**
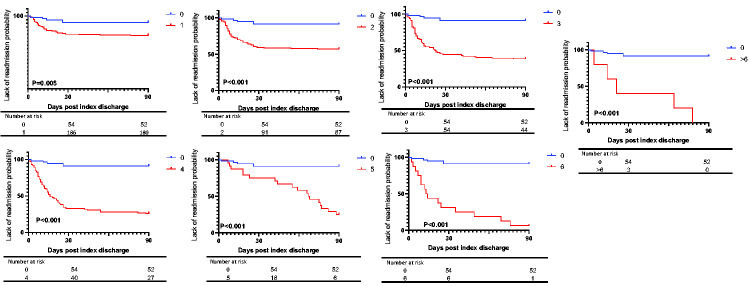
Kaplan–Meier analysis of the CHA_2_DS_2_-VASc score, and 90-day readmission following index admission for atrial fibrillation.

Our study included 717 consecutive hospitalized patients with a primary diagnosis of AF during the study period. Table 1 demonstrates patient demographics stratified by CHA_2_DS_2_-VASc score. Most patients were men (58%) and aged <65 years (65%). The most common comorbidity was hypertension (32%), followed by diabetes (23%), CHF (20%), and ischemic heart disease (20%). The median CHA_2_DS_2_-VASc score was 2.^1–3^ Overall, the median LOS was 2 (IQR: 1–3); in-hospital mortality occurred in 31 patients (4%), with a further 320 patients (45%) readmitted within 90 days of their index hospitalization. The impact of the CHA_2_DS_2_-VASc score on inpatient mortality and LOS is highlighted in Tables 1 and 2, and in Figure 1. Although inpatient mortality increased significantly from 43% to 61% when comparing patients with a score of 2 to those with a score of 3 (P = 0.02), there was no corresponding increase in mortality rates seen with further increases in the CHA_2_DS_2_-VASc score. Furthermore, the logistic regression model did not demonstrate a substantial effect of an increment in CHA_2_DS_2_-VASc score on mortality. There was a progressive increment in LOS from 1 (1–2) day for a CHA_2_DS_2_-VASc of 1 to 5 (4–6) days for a CHA_2_DS_2_-VASc of 6. Furthermore, the regression model showed an increase in IRR starting from a CHA_2_DS_2_-VASc of 2 (IRR: 2, 95% confidence interval [CI]: 1.7–2.2, P = 0.002) to a score of >6 (IRR: 5, 95% CI: 1.8–10.7, P < 0.001), compared with a score of 0. The effect of the CHA_2_DS_2_-VASc on 90-day readmission is illustrated in Table 1 and in Figures 1 and 2. There was a progressively significant increase in the 90-day readmission rate from a CHA_2_DS_2_-VASc score of 0 (9%) to a score of 4 (74%). Compared with a CHA_2_DS_2_-VASc score of 0, there was an incremental rise in the HR from a score of 1 (HR: 23, 95% CI: 1.3–4.1, P = 0.005) to a score of >6 (HR: 41, 95% CI: 31–72, P < 0.001).

**Table 1.** Baseline characteristics and outcomes stratified according to CHA_2_DS_2_-VASc score.

	CHA_2_DS_2_-VASc score								
Variable	Overall (n = 717)	0 (n = 57)	1 (n = 245)	2 (n = 152)	3 (n = 114)	4 (n = 104)	5 (n = 24)	6 (n = 16)	>6 (n = 5)
Demographics, n (%)									
Age <65 years	466 (65%)	61 (100%)	212 (87%)	93 (61%)	65 (57%)	32 (31%)	6 (25%)	1 (6%)	0 (0%)
Age 65–74 years	175 (24%)	0 (0%)	33 (13%)	55 (36%)	34 (30%)	44 (42%)	9 (38%)	0 (0%)	0 (0%)
Age ≥75 years	74 (10%)	0 (0%)	0 (0%)	4 (3%)	13 (15%)	24 (27%)	9 (38%)	15 (94%)	5 (100%)
Male sex	385 (58%)	61 (100%)	138 (56%)	82 (54%)	53 (46%)	34 (33%)	12 (50%)	4 (25%)	0 (0%)
Comorbidities, n (%)									
Hypertension	227 (32%)	0 (%)	37 (15%)	68 (45%)	54 (47%)	39 (38%)	18 (75%)	11 (75%)	2 (40%)
Ischemic heart disease	140 (20%)	0 (%)	31 (13%)	43 (28%)	29 (25%)	25 (24%)	6 (25%)	3 (25%)	3 (60%)
Diabetes mellitus	165 (23%)	0 (%)	29 (12%)	26 (17%)	35 (31%)	46 (44%)	15 (63%)	11 (63%)	5 (100%)
Cerebrovascular disease	137 (19%)	0 (%)	0 (0%)	16 (11%)	35 (31%)	52 (50%)	19 (79%)	10 (79%)	5 (100%)
Peripheral vascular disease	81 (11%)	0 (%)	19 (8%)	1 (1%)	20 (18%)	31 (30%)	5 (21%)	7 (44%)	5 (100%)
Congestive heart failure	141 (20%)	0 (%)	16 (7%)	44 (29%)	38 (33%)	26 (25%)	10 (42%)	5 (31%)	2 (40%)
PCI within the past year	40 (6%)	0 (%)	7 (3%)	8 (5%)	7 (6%)	13 (13%)	2 (8%)	1 (6%)	2 (40%)
CABG within the past year	25 (3%)	0 (%)	7 (3%)	10 (7%)	2 (2%)	1 (1%)	3 (13%)	0 (0%)	1 (20%)
Thyroid disease	34 (5%)	1 (2%)	14 (6%)	8 (5%)	12 (11%)	9 (9%)	5 (21%)	3 (19%)	2 (40%)
Valvular heart disease	120 (17%)	0 (0%)	32 (13%)	21 (14%)	23 (20%)	24 (23%)	4 (17%)	7 (44%)	4 (80%)
Anemia	114 (16%)	2 (4%)	42 (17%)	22 (14%)	27 (24%)	15 (14%)	4 (17%)	0 (0%)	1 (20%)
Dementia	51 (7%)	0 (%)	9 (4%)	11 (7%)	17 (15%)	10 (10%)	3 (13%)	1 (6%)	0 (0%)
Active malignancy	28 (4%)	0 (0%)	9 (4%)	1 (1%)	5 (4%)	9 (9%)	2 (8%)	0 (0%)	1 (20%)
Chronic liver failure	58 (8%)	2 (4%)	20 (8%)	6 (4%)	5 (4%)	12 (12%)	5 (21%)	6 (38%)	2 (40%)
Chronic lung disease	78 (11%)	3 (5%)	34 (14%)	13 (9%)	12 (11%)	9 (9%)	2 (8%)	1 (6%)	0 (0%)
Chronic kidney failure	66 (9%)	4 (7%)	27 (11%)	7 (5%)	6 (5%)	3 (3%)	5 (21%)	4 (25%)	1 (20%)
Outcomes, median (IQR) or n (%)									
LOS of index admission	2 (1–3)	1 (1–2)	2 (1–2)	2 (2–2)	3 (2–4)	3 (3–4)	4 (3–4)	5 (4–6)	5 (5–6)
Mortality during index admission	31 (4%)	2 (4%)	5 (2%)	1 (1%)	7 (6%)	9 (9%)	3 (13%)	2 (13%)	2 (40%)
90-day readmission	320 (45%)	5 (9%)	65 (27%)	66 (43%)	70 (61%)	77 (74%)	18 (75%)	15 (94%)	5 (100%)

PCI, percutaneous coronary intervention; CABG, coronary artery bypass grafting; LOS, length of stay; IQR, interquartile range.

**Table 2.** Role of CHA_2_DS_2_-VASc in predicting 90-day hospital readmission, length of stay, and all-cause inpatient mortality during the index admission.

	Inpatient mortality		90-day readmission		Length of stay	
CHA_2_DS_2_-VASc	aOR (95% CI)	P	HR (95% CI)	P	IRR (95% CI)	P
0	Reference		Reference		Reference	
1	0.57 (0.12–4.1)	0.52	2.3 (1.3–4.1)	0.005	1.2 (0.9–1.5)	0.18
2	0.42 (0.09–1.4)	0.16	3.2 (1.9–5.3)	<0.001	2 (1.7–2.2)	0.002
3	1.2 (0.74–2).	0.45	4.5 (2.8–7.1)	<0.001	2.8 (2.5–3.1)	<0.001
4	1.27 (0.9–2)	0.19	5.6 (3.6–8.7)	<0.001	3.3 (3–3.6)	<0.001
5	1.32 (0.091–1.93)	0.15	20.3 (8.8–32)	<0.001	4 (3.3–4.9)	<0.001
6	1.31 (0.87–1.81)	0.19	38.4 (23.6–65)	<0.001	5 (4–6.2)	<0.001
>6	1.32 (1.05–1.93)	0.06	41 (31–72)	<0.001	5 (1.8–10.7)	<0.001

aOR, adjusted odds ratio; HR, hazard ratio; IRR, incident rate ratio; CI, confidence interval.

Models were adjusted for covariate factors such as chronic kidney disease, valvular heart disease, and thyroid disease.

## Discussion

This was the first study to describe the utility of the CHA_2_DS_2_-VASc score in predicting inpatient LOS and 90-day readmission following the index admission in patients with a primary diagnosis of AF in Syria. This study highlights multiple important novel findings for the Arabic population. First, nearly half of the patient cohort was readmitted within 90 days following their index admission. Second, the CHA_2_DS_2_-VASc score was positively correlated with LOS during the index admission as well as the 90-day readmission rate. Third, the CHA_2_DS_2_-VASc score was not associated with inpatient mortality during the index admission in our cohort.

AF has enormous impacts on economies worldwide.^16^ Recent studies have focused on many aspects of AF, including hospitalization and readmission rates.^14,17^ Unsurprisingly, our 90-day readmission rate of 45% was significantly higher than the reported rate in the US National Registry 2013 of 17.6% to 25.1%.^14,18^ There are no data from the developing world to compare with the current dataset. Our higher readmission rates may partially be attributed to the conflict in Syria, which has been ongoing since 2011 and has massively affected health infrastructure. This situation has resulted in a high turnover of skilled staff and insufficient numbers of nurses and allied health professionals.^19^ Only half of Syria’s hospitals and primary health care centers are fully functional,^19^ making it difficult to follow patients who present to hospitals with acute AF after discharge and manage their risk factors. Although there are no data prior to the conflict for comparison, the current data are likely reflective of the current state in this war-torn country. Our data suggest that increased support for the Syrian health care system, especially primary care, may help to reduce the 90-day readmission rate in Latakia and nationwide. Furthermore, socioeconomic disparities may affect the ability of patients to adhere to management plans and attend follow-up appointments. Financial constraints may also limit access to necessary interventions and medications.

AF is a strong risk factor for thromboembolic events. The CHA_2_DS_2_-VASc score was developed to predict vascular events and stroke and to guide anticoagulation treatment in patients with AF.^20^ The constituents of this scoring system, including CHF, hypertension, age ≥75 years, type 2 diabetes, previous transient ischemic attack or stroke or thromboembolism, sex, and vascular disease, have all been proven to be independent risk factors for adverse cardiovascular outcomes.^20^ The score has been used in subsequent studies to predict outcomes in patients with and without AF, including patients with myocardial infarction^21,22^ and AF ablation.^23^ The positive correlation of the CHA_2_DS_2_-VASc score with LOS and 90-day readmission was in keeping with previous studies from developed countries.^14^ The patient’s health status primarily influences systemic health care factors and the nature of their illness. A higher CHA_2_DS_2_-VASc score reflects increased cardiovascular morbidity and potentially decreased physiological reserve, contributing to the increased LOS. Similarly, the increased cardiovascular disease burden characterized by a higher CHA_2_DS_2_-VASc score was found to be associated with a higher readmission rate, in keeping with a previous study in a US-based cohort suggesting that older patients with increased cardiovascular comorbidities were more likely to be readmitted.^24^ Our study findings demonstrated a significant association of higher CHA_2_DS_2_-VASc scores with an increased risk of 90-day hospital readmission and LOS in patients with AF. This aligns with previous research indicating the utility of the CHA_2_DS_2_-VASc score in predicting adverse outcomes and resource utilization across various cardiovascular populations, including patients with AF.^14^ For instance, a large database study assessing outcomes following coronary artery bypass grafting showed that higher CHA_2_DS_2_-VASc scores were associated with increased 90-day readmission, longer LOS, and higher health care costs in AF and non-AF populations.^25^ These findings highlight the score’s ability to stratify risk beyond its original role in thromboembolic risk assessment.^25^ Similarly, a study evaluating patients post-cardioversion for AF demonstrated a significant relationship between CHA_2_DS_2_-VASc scores and the risk of thromboembolic complication-related readmission within 90 days, further corroborating its prognostic utility in predicting post-discharge outcomes.^26^

The biological mechanisms underlying the association between CHA_2_DS_2_-VASc scores and adverse outcomes likely stem from the score’s representation of cumulative cardiovascular comorbidity. Components of the score, such as CHF, hypertension, and diabetes, are independently linked to chronic inflammation, endothelial dysfunction, and hemodynamic instability, contributing to longer hospital stays and higher readmission rates.^27,28^ Additionally, patients with higher scores often have reduced physiological reserves and are at greater risk of complications during recovery, necessitating repeat hospitalizations.

From a clinical perspective, our findings highlight the CHA_2_DS_2_-VASc score as a practical tool for risk stratification in diverse clinical scenarios. Identifying patients with higher scores allows for targeted interventions, such as intensive post-discharge monitoring, tailored management of comorbidities, and patient education, which could mitigate readmissions and improve patient outcomes. In resource-limited settings, such as Syria, this tool offers a cost-effective strategy for prioritizing care and optimizing resource allocation.

Emerging evidence highlights the prognostic role of the CHA_2_DS_2_-VASc score in non-AF populations, particularly among patients with CHF. A previous study demonstrated that the CHA_2_DS_2_-VASc score independently predicted CHF hospitalizations and a combined endpoint of CHF hospitalizations and all-cause mortality over a 30-month follow-up.^29^ Additionally, a large Danish cohort study demonstrated that the CHA_2_DS_2_-VASc score could predict thromboembolic events, ischemic stroke, and death in patients with CHF regardless of AF status, with a higher absolute risk observed in patients without AF who had elevated scores.^30^ These findings emphasize the value of the CHA_2_DS_2_-VASc score in stratifying risk across CHF subgroups. Further supporting this, a study of more than 7000 patients with CHF revealed a consistent relationship between increased CHA_2_DS_2_-VASc scores and adverse outcomes, including cardiac hospitalizations and death, irrespective of ejection fraction.^11^ Moreover, in older patients with CHF, a CHA_2_DS_2_-VASc score ≥5 was independently associated with higher all-cause mortality and rehospitalization risk over mid-term follow-up, irrespective of AF presence, underscoring its broad clinical applicability.^10^ Together, these findings demonstrate that the CHA_2_DS_2_-VASc score reflects a cluster of cardiovascular risk factors that significantly impact prognosis regardless of AF status. Implementing this score in clinical practice could enhance risk stratification and inform management decisions across diverse patient populations.

## Limitations

The inherent biases of retrospective studies, including reliance on medical records, could have influenced data completeness and accuracy. Data collection was limited to a single tertiary care center in Latakia. This city was relatively less affected by the Syrian conflict than other northern and eastern regions of Syria; therefore, our results might not be generalizable to other centers/regions given the significant heterogeneity in the quality and level of hospital supplies and staffing. Additionally, our analysis included only routinely collected data within the medical records and according to the number of patients who presented to the hospital; therefore, other variables potentially impacting LOS, 90-day readmission, and inpatient mortality may have been excluded. The study did not address treatments given during the index admission, which could have affected the study outcomes. Additionally, although sufficient for initial analyses, the sample size may limit the statistical power for detecting smaller effect sizes or more complex interactions. Future research should validate these findings in larger, multicenter studies that include diverse populations and health care settings. Investigating the predictive value of the CHA_2_DS_2_-VASc score in non-AF populations and its role in specific subgroups, such as older patients or those with comorbid heart failure, could further support its clinical utility. Prospective studies incorporating comprehensive follow-up and interventions targeting high-risk groups identified using the CHA_2_DS_2_-VASc score would provide robust evidence to guide clinical practice. Moreover, exploring the score’s application in other conflict or resource-limited environments could offer valuable insights into its adaptability and scalability.

Despite these limitations, our study underscores the potential of the CHA_2_DS_2_-VASc score as a simple and effective tool for risk stratification, enabling more efficient resource allocation and improved patient outcomes in challenging health care settings.

## Conclusion

The CHA_2_DS_2_-VASc score represents a group of risk factors that can be used as surrogate markers of a patient’s risk profile for predicting outcomes beyond the risk of thromboembolic stroke in patients with AF. Encouraging Syrian physicians to apply the CHA_2_DS_2_-VASc score to assess hospital readmission risk can help in delivering effective preventative treatments and improve patient care in conflict settings. Further data from the developing world is needed to help optimize outcomes in communities with limited resources.
